# Fast detection of deletion breakpoints using quantitative PCR

**DOI:** 10.1590/1678-4685-GMB-2015-0159

**Published:** 2016-06-16

**Authors:** Gulshara Abildinova, Zhanara Abdrakhmanova, Helena Tuchinsky, Elimelech Nesher, Albert Pinhasov, Leon Raskin

**Affiliations:** 1 National Research Center of Maternal and Child Health, Astana, Kazakhstan; 2 Department of Molecular Biology, Ariel University, Ariel, Israel; 3 Department of Medicine, Vanderbilt University, Nashville, TN, USA

**Keywords:** deletion boundaries, deletion breakpoints, DMD gene, Duchenne and Becker muscular dystrophies, hemizygous deletions, heterozygous deletions

## Abstract

The routine detection of large and medium copy number variants (CNVs) is well established. Hemizygotic deletions or duplications in the large Duchenne muscular dystrophy *DMD* gene responsible for Duchenne and Becker muscular dystrophies are routinely identified using multiple ligation probe amplification and array-based comparative genomic hybridization. These methods only map deleted or duplicated exons, without providing the exact location of breakpoints. Commonly used methods for the detection of CNV breakpoints include long-range PCR and primer walking, their success being limited by the deletion size, GC content and presence of DNA repeats. Here, we present a strategy for detecting the breakpoints of medium and large CNVs regardless of their size. The hemizygous deletion of exons 45-50 in the *DMD* gene and the large autosomal heterozygous *PARK2* deletion were used to demonstrate the workflow that relies on real-time quantitative PCR to narrow down the deletion region and Sanger sequencing for breakpoint confirmation. The strategy is fast, reliable and cost-efficient, making it amenable to widespread use in genetic laboratories.

## Introduction

The number of reported single nucleotide variants and small indels has grown significantly since completion of the Human Genome Project (Naidoo *et al.*, 2011). However, the list of medium and large germline insertions, deletions, inversions and translocations is far from complete (MacDonald *et al.*, 2014). The Database of Genomic Variants (MacDonald *et al.*, 2014) established to catalogue copy number variations (CNVs) larger than 50 bp contains millions of CNVs with median size alterations of 1-10 kb. For the majority of CNVs, the database provides the confidence interval where the breakpoints likely reside, but no exact deletion or insertion breakpoints are known. The identification of CNV breakpoints may be of great importance in research and clinical diagnosis. For instance, variation in the size of a deletion in Williams syndrome involves different genes that contribute to distinct phenotypes of this multisystem disorder (Ibn-Salem *et al.*, 2014).

The routine detection of large deletions is well established in genetic laboratories, with the spectrum of methods for deletion analysis ranging from relatively inexpensive techniques such as multiple ligation probe amplification (MLPA) to more time- and resource-consuming approaches such as whole-genome sequencing. MLPA has limited resolution in that it does not provide a specific location of the deletion breakpoints and does not discriminate among deletions of different sizes involving the same exons. While array-based comparative genomic hybridization (aCGH) is considered a gold standard for CNV analysis, high-density single nucleotide polymorphism (SNP) genotyping arrays used for aCGH analysis also do not provide a definite breakpoint location. The resolution of next generation sequencing is comparable to aCGH, but the method is still prohibitively expensive and CNV calling algorithms are not yet optimal (Hayes *et al.*, 2013). The identification of deletion breakpoints can help to genotype the family members of a deletion carrier, shed light on the mechanisms of deletion and predict deleterious mutations associated with the disease.

Duchenne and Becker muscular dystrophies (DMD and BMD) are the most common pediatric neuromuscular disorders (Mathews *et al.*, 2010; Liew and Kang, 2013; Ness and Apkon, 2014). These disorders are caused by mutations in the *DMD* gene encoding dystrophin that is involved in the maintenance of muscle cell membranes (Blake *et al.*, 2002; del Gaudio *et al.*, 2008). *DMD* is one of the largest human genes and spans 2.4 Mb on chromosome X. The majority of *DMD* mutations involve a hemizygous deletion or duplication of one or more exons, while about a third of the mutations are formed *de novo* (Santos *et al.*, 2014). The deletion of exons 45-55 of the *DMD* gene has received special attention because it is associated with a milder phenotype of the disorder (Miyazaki *et al.*, 2009). MLPA designed to detect medium size deletions or duplications is generally used for mutation testing in *DMD*, but only provides information about deleted exons, with the exact location of the deletion breakpoints in large introns remaining undefined.

Methods commonly used to detect deletion boundaries include long-range PCR and primer walking (Quadri *et al.*, 2015). However, the success of these methods depends on many different factors, including deletion size, GC content and the presence of DNA repeats. There is therefore a need for a fast, simple, reliable and cost-efficient method for detecting the breakpoints of medium and large deletions. In this report, we describe a strategy to detect CNV breakpoints meeting the foregoing criteria and demonstrate its applicability by using a hemizygous *DMD* deletion and an autosomal heterozygous *PARK2* deletion as examples. Our strategy requires only quantitative PCR for breakpoint detection and Sanger sequencing for confirmation of the findings. These characteristics should make this strategy accessible to many genetics laboratories.

## Materials and Methods

### DNA isolation and real-time quantitative PCR

Genomic DNA was extracted from blood samples by using a Puregene DNA extraction kit (Gentra Systems) according to the manufacturer's protocol. Primers were designed using Primer 3 software and synthesized by Integrated DNA technologies (Coralville, IA, USA). The primers used are listed in Supplementary Tables S1-S3. Real-time quantitative PCR (qPCR) was done using an MxPro3000 apparatus (Stratagene, Santa Clara, CA, USA) and an Applied Biosystems ABI 7900HT thermocycler (Applied Biosystems, Foster City, CA), with the following thermal profile: 180 s at 95 °C, followed by 40 cycles of 3 s at 95 °C and 30 s at 60 °C. The specificity of each primer set was monitored by dissociation curve analysis using the following profile: 30 s at 95 °C, 30 s at 55 °C and 30 s at 95 °C. The reactions were done in duplicate using a SYBR Fast Universal Readymix kit (KAPA, Woburn, MA, USA), 125 nM of forward and reverse primers, and 10 ng of DNA.

### MLPA analysis

DNA samples from a healthy farther and his two sons diagnosed with DMD were used in this study. MLPA kits (SALSA MLPA P034 DMD mix 1 and SALSA MLPA P035 DMD mix 2) for *DMD* and *BMD* analysis were used according to the manufacturers protocol (MRC-Holland, Amsterdam, The Netherlands). Briefly, DNA (20 ng) was denatured and fragmented for 5 min at 98 °C was followed by MLPA probe hybridization for 16 h at 60 °C and ligation for 15 min at 54 °C. After PCR amplification of the ligation with fluorescently labeled primer the product was used for fragment analysis in a 3500 Series Genetic Analyzer (Applied Biosystems). Fragment analysis was done using GeneMarker 2.4.0 (Softgenetics, State College, PA). The molecular diagnosis of DMD was established using MLPA at the National Research Center of Child and Maternal Health in Astana, Kazakhstan.

### Sanger sequencing

PCR primer pairs (forward: 5'-GCTGTGGGTGAAAATGCCTT-3' and reverse: 5'-TGAAGGGACATTGGAGATTG-3') were used to amplify the region containing the breakpoint between exon 44 and exon 51 caused by a deletion in the *DMD* gene. Each PCR reaction contained Ampli*Taq* Gold^®^ 360 PCR Master Mix (Applied Biosystems), 10 μM primers and 50-100 ng of template gDNA/μL in a final volume of 25 μL. The cycling conditions were: 95°C 10 min, 35 cycles of 95 °C for 15 s, 62 °C for 3 s and 72 °C for 60 s, and a final extension at 72 °C for 7 min. The quality of the amplified products was assessed using agarose gel electrophoresis and the PCR fragment was extracted from the gel using a Qiagen Gel extraction protocol (QIAquick^®^ gel extraction kit; Qiagen, Valencia, CA). Clean PCR product was used for Sanger sequencing in a 3500 Series Genetic Analyzer.

## Results

### MLPA diagnosis of DMD

**Figure 1:**
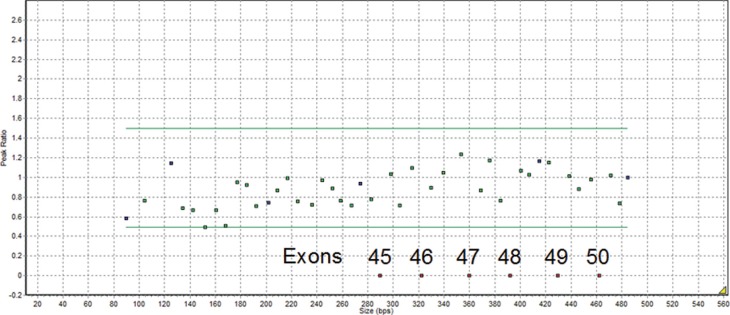
MLPA results showing the deletion of exons 45-50 in the *DMD gene.*

MLPA analysis of two boys (6 and 8 years old) with symptoms of DMD identified a deletion between exons 44 and 51 of the *DMD* gene that was characterized by the absence of the probe signal at the expected positions for exons 45-50 (Figure 1).

### Narrowing down the region of the deletion

**Figure 2:**
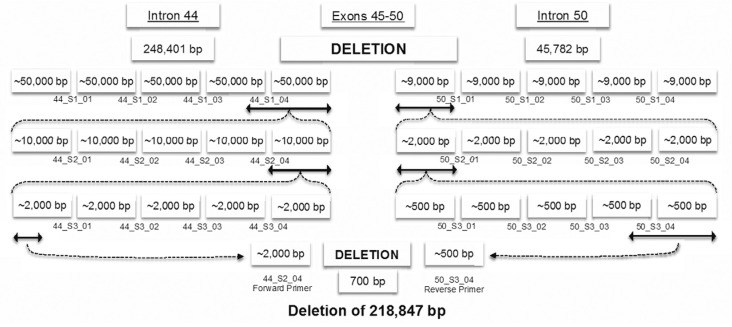
Strategy for breakpoint detection of the *DMD* exon 45-50 deletion. The figure shows narrowing down of the deletion region in three steps using real-time qPCR and confirmation of the connection point of the deletion using Sanger sequencing.

Since exons 45 to 50 were deleted, we assumed that the breakpoints of the deletion were located in the introns between exons 44 and 45 at the 5' end and between exons 50 and 51 at the 3' end. Use of the Ensembl Genome browser (http://useast.ensembl.org) revealed that intron 44 contained 248,401 bp and intron 50 contained 45,782 bp (transcript ENST00000357033) (Figure 2). This finding indicated that the region containing the breakpoint between exons 44 and 51 could not be more than 294,183 bp in size (from the 5' end of exon 45 to the 3' end of exon 50). We used sets of primers to divide these regions into equal size fragments (Figure 2). Four sets of primers were designed to divide intron 44 to five fragments that spanned approximately 50,000 bp each (Table S1). Similarly, four sets of primers were designed to divide intron 50 into five fragments that spanned about 9,000 bp each (Table S2). In both regions (introns 44 and 50), three distal sets of primers amplified products in the normal (father) and mutant (sons) DNA samples, while proximal sets in both introns were not amplified in either of the mutant samples (Figure 2).

Based on these results, we concluded that the deletion was located between the reverse primer from set 44_S1_04 that did not show amplification and the reverse primer of the closest set 44_S1_03 that did show amplicon formation (Figure 2). This finding allowed us to reduce the potential region of the deletion five-fold. By using this approach, we narrowed down the potential region of interest containing the breakpoint between exons 44 and 51 from 294,183 bp to approximately 2,500 bp (Figure 2). This region was amplified by using the forward primer from the proximal primer set that showed amplification in intron 44 (44_S4_forward) and the reverse primer from the proximal primer set that showed amplification in intron 50 (50_S4_reverse) (Figure 2; Table S3). Real-time qPCR using this set of primers resulted in amplification in the mutants (sons) and no amplification in the normal sample (father).

### Confirmation of the deletion breakpoints

**Figure 3:**
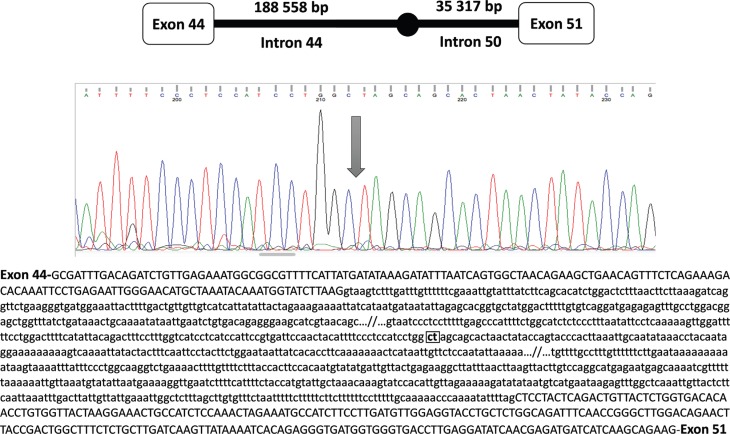
The *DMD* exon 45-50 deletion breakpoint determined by Sanger sequencing.

The real-time qPCR results were confirmed by gel electrophoresis that showed an amplicon of 700 bp in the *DMD* mutant samples. Sanger sequencing of this amplicon using the same primers (44_S4_forward and 50_S4_reverse) identified the deletion breakpoints (Figure 3). The size of the deletion from intron 44 to intron 50 of the *DMD* gene was 218,847 bp.

### Applicability for the detection of heterozygous deletion breakpoints

To evaluate the usefulness of our strategy for identifying heterozygous autosomal deletion breakpoints, we analyzed a large heterozygous deletion in the *PARK2* gene of a mother and her son. The deletion on chromosome 6 was initially detected by SNP microarray and included a fragment of 466,304 bp located between two probes. The deletion included the *PARK2* gene but the exact location of the breakpoints was unknown. The boy's father did not have the deletion and his DNA was used as a reference.

We applied the strategy used for *DMD* analysis to identify the heterozygous deletion by using several qPCR probe sets in triplicate, with the *GAPDH* gene as a reference. The qPCR curves of the wild-type homozygotes and heterozygous deletion carriers were clearly distinct (Figure S1). The analysis reduced the size of the unknown fragment to a manageable 1,106 bp. Sanger sequencing of the fragment showed the deletion of 383,218 bp (chr6: 162,215,242 −162,598,460 in genome GRCh38.p2) that was replaced with a 26 bp insertion (Figure S2). The deletion extended from intron 1 to intron 3 and included exons 2 and 3.

## Discussion

The identification of CNV breakpoints has always been a challenging undertaking. Many studies that identified large deletions or insertions limited their analysis to the discovery itself because searching for the exact breakpoints of these mutations would be expensive and time-consuming. Identification of the breakpoints of novel germline deletions or insertions could provide information on the involvement of a specific gene in the pathogenesis of a hereditary condition and simplify mutation detection within the carrier's family. Some studies have used real-time qPCR to demonstrate the presence of a deletion (Stittrich *et al.*, 2014). In this work, we utilized real-time qPCR to develop a new strategy that allows straightforward, reliable identification of CNV breakpoints in germline DNA, regardless of the size of the deletion.

We demonstrated the usefulness of our strategy by identifying the breakpoints of a hemizygotic deletion in exons 45-50 of the *DMD* gene. Mutations in this gene are routinely detected using aCGH, multiplex PCR, Southern blotting and MLPA, but these methods are unable to determine the precise breakpoints of deletions and duplications. Deletions in exons 44-55 of the *DMD* gene have received special attention because of the mild phenotype associated with them. As also observed in other studies (Miyazaki *et al.*, 2009), we found no repetitive sequences or significant homology between the sequences adjacent to the deletion breakpoints. However, we found two identical 15-bp fragments located 1,121 bp 5' of the deletion in intron 44 and 428 bp 3' of the deletion in intron 50. This fragment may be involved in a recombination event that led to the deletion of exons 45-50. Since the use of our strategy to detect CNV breakpoints in a hemizygotic deletion in the *DMD* gene could be considered to be a rather special case that may not reflect the general applicability of the method, we also used this approach to detect the breakpoint in autosomal heterozygous deletions. Real-time qPCR has previously been used to successfully detect heterozygous deletions (Zinke *et al.*, 2015).

While the exact cost of our strategy for identifying CNV breakpoints will depend on the size of the CNV and cost of consumables, the analysis requires only the availability of oligonucleotides, PCR reagents and Sanger sequencing, which should make this strategy affordable to many laboratories. The high reliability of the method makes it possible to calculate the upfront costs of the analysis for a specific deletion.

In conclusion, the strategy described here provides a reliable, relatively inexpensive and fast method for identifying the breakpoints of medium and large CNVs in *DMD* and autosomal genes. Next-generation sequencing may replace this method in the future, however sequencing of an entire gene, e.g., the *DMD* gene that includes large introns, remains expensive.

## Supplementary Material

The following online material is available for this article:

Table S1 - Primer sets for narrowing down the region of the deletion in 5' (intron 44).

Table S2 - Primer sets for narrowing down the region of the deletion in 5' (intron 50).

Table S3 - Primers for detecting the connection point of the deletion by real-time qPCR and Sanger sequencing.

Figure S1 - Detection of the heterozygous deletion in PARK2 by real-time qPCR.

Figure S2 - Sanger sequencing of the PARK2 deletion.
