# Supplementary material for: Fast detection of deletion breakpoints using quantitative PCR
Source: Genet Mol Biol. 2016 Jun 16;39(3):365–9. doi: 10.1590/1678-4685-GMB-2015-0159 (PMC5004823; doi:10.1590/1678-4685-GMB-2015-0159)
Supplement: Supplementary file 1 [file 1415-4757-gmb-1678-4685-GMB-2015-0159-Suppl01.pdf]

Table S1 - Primer sets for narrowing down the region of the deletion in 5’ (intron 44).

Name	Primer	Sequence	Product size (bp)	Amplicon
44_S1_01	Forward	cccatgttgagggacaaaag	149	Yes
	Reverse	attttcttcccatcccacct		
44_S1_02	Forward	gcctacgattccaccacaaa	236	Yes
	Reverse	ggtgagattggatggtttgg		
44_S1_03	Forward	gccctgggatgatctaagct	184	Yes
	Reverse	aggtcaggcgtttgagatca		
44_S1_04	Forward	ggacacagaaagaggcctca	199	No
	Reverse	tcgcccaattcacagcattt		
44_S2_01	Forward	tccgttgcaactgttgtctg	150	Yes
	Reverse	tgtgtatcaacggcagtgtg		
44_S2_02	Forward	tgcttctcccacttggcata	222	Yes
	Reverse	caaatgtgcaggtccggaaa		
44_S2_03	Forward	gagtggaaactgctttgggg	176	Yes
	Reverse	tgtgtgtaagcgtgtgtgtg		
44_S2_04	Forward	tcacttgcttatgatgccgc	200	Yes
	Reverse	aaggcattttcacccacagc		
44_S3_01	Forward	agctttcccctaccatcgag	222	No
	Reverse	gagccagtggtgaagagact		
44_S3_02	Forward	gtagccctggagtctttgga	244	No
	Reverse	tgtccaccaaaccagtttaca		
44_S3_03	Forward	gttcccttcctccctccttc	246	No
	Reverse	ggataaagaatgtggcgcgt		
44_S3_04	Forward	tgttggacagcacaggttta	152	No
	Reverse	ggttacttttggctctgggg		
