# Supplementary material for: Fast detection of deletion breakpoints using quantitative PCR
Source: Genet Mol Biol. 2016 Jun 16;39(3):365–9. doi: 10.1590/1678-4685-GMB-2015-0159 (PMC5004823; doi:10.1590/1678-4685-GMB-2015-0159)
Supplement: Supplementary file 2 [file 1415-4757-gmb-1678-4685-GMB-2015-0159-Suppl03.pdf]

Table S3 - Primers for detecting the connection point of the deletion by real-time qPCR and Sanger sequencing.

Name	Primer	Sequence	Product size (bp)	Amplicon
44_S4	Forward	gctgtgggtgaaaatgcctt	No more than 1241	DMD only
50_S4	Reverse	tgaagggacattggagattg		
