# Supplementary material for: Fast detection of deletion breakpoints using quantitative PCR
Source: Genet Mol Biol. 2016 Jun 16;39(3):365–9. doi: 10.1590/1678-4685-GMB-2015-0159 (PMC5004823; doi:10.1590/1678-4685-GMB-2015-0159)
Supplement: Supplementary file 3 [file 1415-4757-gmb-1678-4685-GMB-2015-0159-Suppl02.pdf]

**Table S2 -** Primer sets for narrowing down the region of the deletion in 5’ (intron 50).

| Name | Primer | Sequence | Product size (bp) | Amplicon |
|---|---|---|---|---|
| 50_S1_01 | Forward | ggccaggtccttcaacaatg | 174 | No |
| | Reverse | agagagggagaagaaagggaac | | |
| 50_S1_02 | Forward | aaagacgctccttacctccc | 173 | Yes |
| | Reverse | gctaggtaaggaggaggcag | | |
| 50_S1_03 | Forward | agcactgtaacacaccctct | 161 | Yes |
| | Reverse | gcaacaggggtgccttattt | | |
| 50_S1_04 | Forward | ctccgtcttttccagctgtg | 161 | Yes |
| | Reverse | ggggtagcttgttacacagc | | |
| | | | | |
| 50_S2_01 | Forward | cctttgaacctgcacactgt | 158 | Yes |
| | Reverse | ttgtctttcagctggtggga | | |
| 50_S2_02 | Forward | gcatgcttcttggctggaat | 179 | Yes |
| | Reverse | tccttgagtgtgcatccctt | | |
| 50_S2_03 | Forward | ggattactgtgtgccctcct | 196 | Yes |
| | Reverse | ctctgacatcgctctgttgc | | |
| 50_S2_04 | Forward | tcatccaggccctgttttca | 201 | Yes |
| | Reverse | tcaggggaaggtcatgcatt | | |
| | | | | |
| 50_S3_01 | Forward | accctaccgctcaaaatgga | 220 | No |
| | Reverse | gagcctttcctgagatagtgga | | |
| 50_S3_02 | Forward | tttaacgtctctgtggccct | 177 | No |
| | Reverse | gacacacctggtatttggca | | |
| 50_S3_03 | Forward | accctcattcttgactgtgtc | 174 | No |
| | Reverse | tgtctaatgaactcagcatggg | | |
| 50_S3_04 | Forward | agccaatctccaatgtccct | 238 | Yes |
| | Reverse | aggagttctgttttgatcatgct | | |
