# Supplementary material for: Fast detection of deletion breakpoints using quantitative PCR
Source: Genet Mol Biol. 2016 Jun 16;39(3):365–9. doi: 10.1590/1678-4685-GMB-2015-0159 (PMC5004823; doi:10.1590/1678-4685-GMB-2015-0159)
Supplement: Supplementary file 4 [file 1415-4757-gmb-1678-4685-GMB-2015-0159-Suppl04.pdf]

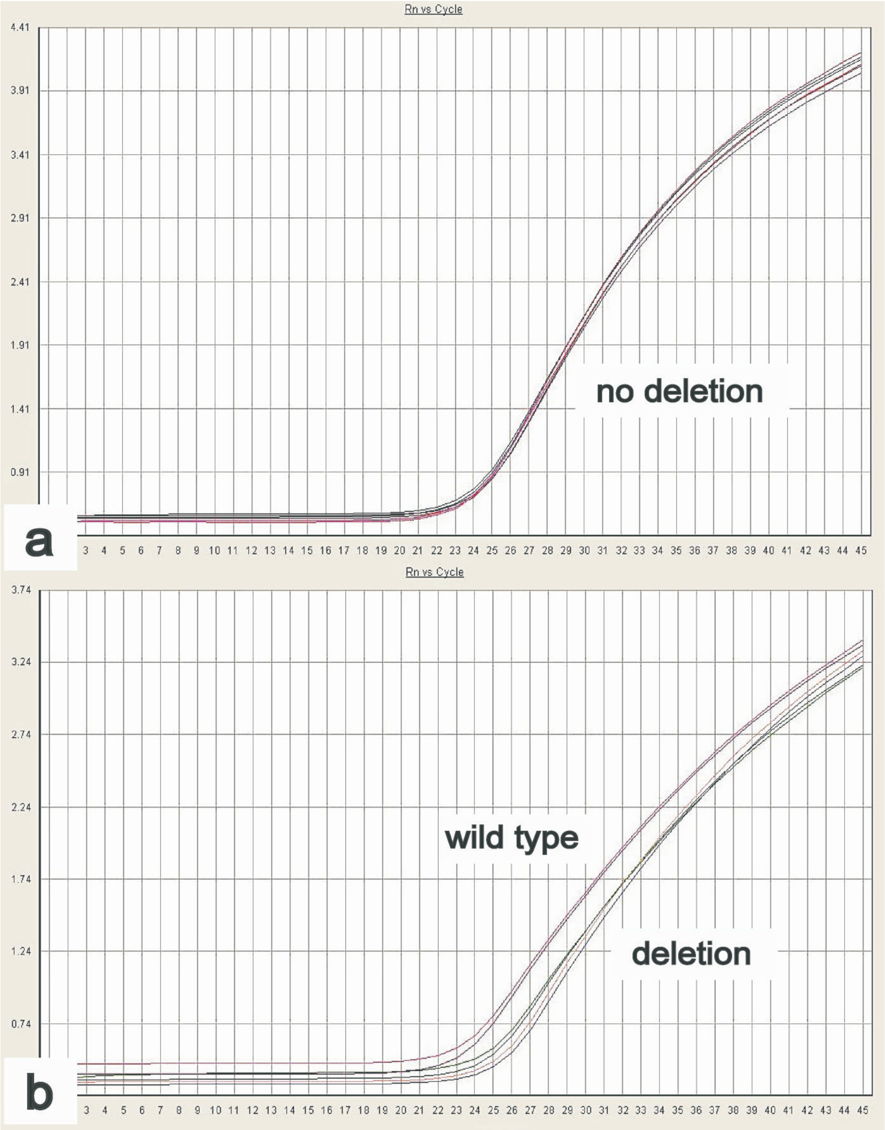

Figure S1 - Detection of the heterozygous deletion in PARK2 by real-time qPCR. (a) Real-time qPCR curves showing simultaneous amplification of the fragment without deletion and (b) delayed amplification of the fragment with the heterozygous deletion as compared to the wild type.
