# Supplementary material for: Fast detection of deletion breakpoints using quantitative PCR
Source: Genet Mol Biol. 2016 Jun 16;39(3):365–9. doi: 10.1590/1678-4685-GMB-2015-0159 (PMC5004823; doi:10.1590/1678-4685-GMB-2015-0159)
Supplement: Supplementary file 5 [file 1415-4757-gmb-1678-4685-GMB-2015-0159-Suppl05.pdf]

PARK2 gene from Intron 1 to Intron 3, Exons 2 and 3 are deleted

GRCh38.p2chr6:162,215,243 -162,598,458 (***383,215 bp***) deleted, and replaced with 26 nucleotides:
***GGAAGCTTGAAGGCTACATTTTGGAA**

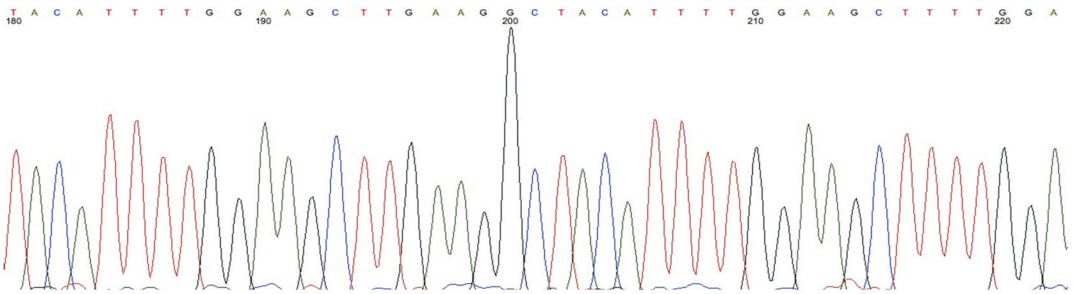

Intron 3-cttccccatctgcaaccttattgggcccagaaataccacttcctctaggaagtcttacttaattcctccatttaatgtaaatcccctcccccctaaatgcccactacatttgagtt gcatcattcttaggggtcgtaagctacattctatctcacaatatcccatagataattttatctctcctaccaggctttagattcttttgaaggtgaaggctacatttt/*........./ccagtttcc atttctggacatatgatcaattaaactgtaatccttcctgctacaaaacaccagaaaagctggctcggtgataagaagcattttcaaatgttgaagtcatttataaaaagaaatccccag gcagttaagaaacaatgagtggacacaaaataagagctaacccagaagataatgacacttcccctaaattggctagtcccggcaaccagtaggcttgaaagctgtgccctcaaaagg ctgtggccttgatggtggaccaggaaagtccacccgacctcacaagcaggacaggttgaatttgcgtgtcttttgtccag-**Intron 1**

Figure S2 - Sanger sequencing of the PARK2 deletion. Sanger sequencing of the fragment showed the deletion of 383,218 bp (chr6: 162,215,242 – 162,598,460 in genome GRCh38.p2) replaced with a 26 bp insertion. The deletion extended from intron 1 to intron 3 and included exons 2 and 3.
